# Genetic and pathogenic potential of highly pathogenic avian influenza H5N8 viruses from live bird markets in Egypt in avian and mammalian models

**DOI:** 10.1371/journal.pone.0312134

**Published:** 2024-10-29

**Authors:** Yassmin Moatasim, Basma Emad Aboulhoda, Mokhtar Gomaa, Ahmed El Taweel, Omnia Kutkat, Mina Nabil Kamel, Mohamed El Sayes, Mohamed GabAllah, Amany Elkhrsawy, Hend AbdAllah, Ahmed Kandeil, Mohamed Ahmed Ali, Ghazi Kayali, Rabeh El-Shesheny

**Affiliations:** 1 Center of Scientific Excellence for Influenza Viruses, National Research Centre, Giza, Egypt; 2 Department of Anatomy and Embryology, Faculty of Medicine, Cairo University, Cairo, Egypt; 3 Department of Infectious Diseases, St. Jude Children’s Research Hospital, Memphis, TN, United States of America; 4 Human Link DMCC, Dubai, United Arab Emirates; Uppsala University, SWEDEN

## Abstract

Since its first isolation from migratory birds in Egypt in 2016, highly pathogenic avian influenza (HPAI) H5N8 has caused several outbreaks among domestic poultry in various areas of the country affecting poultry health and production systems. However, the genetic and biological properties of the H5N8 HPAI viruses have not been fully elucidated yet. In this study, we aimed to monitor the evolution of circulating H5N8 viruses and identify the pathogenicity and mammalian adaptation *in vitro* and *in vivo*. Three H5N8 HPAI viruses were used in this study and were isolated in 2021–2022 from poultry and wild birds during our routine surveillance. RNA extracts were subjected to full genome sequencing. Genetic, phylogenetic, and antigenic analyses were performed to assess viral characteristics and similarities to previously isolated viruses. Phylogenetic analysis showed that the hemagglutinin genes of the three isolates belonged to clade 2.3.4.4b and grouped with the 2019 viruses from G3 with high similarity to Russian and European lineages. Multiple basic amino acids were observed at cleavage sites in the hemagglutinin proteins of the H5N8 isolates, indicating high pathogenicity. In addition, several mutations associated with increased virulence and polymerase activity in mammals were observed. Growth kinetics assays showed that the H5N8 isolate is capable of replicating efficiently in mammalian cells lines. *In vivo* studies were conducted in SPF chickens (White Leghorn), mice, and hamsters to compare the virological characteristics of the 2022 H5N8 isolates with previous H5N8 viruses isolated in 2016 from the first introduction. The H5N8 viruses caused lethal infection in all tested chickens and transmitted by direct contact. However, we showed that the 2016 H5N8 virus causes a higher mortality in chickens compared to 2022 H5N8 virus. Moreover, the 2022 virus can replicate efficiently in hamsters and mice without preadaptation causing systemic infection. These findings underscore the need for continued surveillance of H5 viruses to identify circulating strains, determine the commercial vaccine’s effectiveness, and identify zoonotic potential.

## Introduction

Highly pathogenic avian influenza (HPAI) viruses are circulating in domestic poultry in many countries worldwide causing huge economic losses. HPAI H5 viruses were first detected in 1959 in Scotland and were isolated from chickens [1]. Infections with HPAI were mainly restricted to poultry till 1996 when the HPAI A/goose/Guangdong/1/96 (H5N1) virus was detected in an outbreak in domestic geese in Guangdong province with unusual high pathogenic effects in infected domestic avian and wild birds [2]. This virus gained the ability to infect humans for the first time through direct contact with infected birds [2]. Since then, this lineage of Gs/Gd/1/96 HP H5 has evolved into ten clades (0 to 9) and then into subclades [3].

Waterfowl (Anseriformes) and shorebirds (Charadriiformes) serve as the main reservoir of influenza A viruses. Over the last two decades, the world witnessed multiple intercontinental epidemic waves of the HPAI [4–7]. In 2008, clade 2.3.4 was first detected in China and evolved into subclades till the detection of the clade 2.3.4.4 group b in 2014 in China and Korea which then spread worldwide along migratory flyways by waterfowl by the end of 2016 [8, 9]. Clade 2.3.4.4 viruses showed high ability of reassorting with different co-circulating virus neuraminidases to form different subtypes such as H5N1, H5N2, H5N5, H5N6, and H5N8 [10–12]. Extensive reassortment events in their viral internal genes caused many genetic constellations of H5NX that co-circulated simultaneously in many countries to be introduced into domestic poultry [11, 13, 14]. The persistent circulation of clade 2.3.4.4 viruses and their rapid evolution lead to the emergence of the highly pathogenic A/(H5N1) clade 2.3.4.4b virus that was detected in wild and domestic poultry in Africa, Asia, and Europe causing massive mortality in infected birds in 2020–2021 [15]. This virus has then spread in animal populations in the Americas since 2021 [6, 16].

Clade 2.3.4.4b showed mammalian adaptation genetic markers that enhance transmission, replication, polymerase activity, and viral binding to the mammalian-like sialic acid α2,6 gal receptors, and was identified and isolated from various mammalian species and humans [17–19]. Several spillover events of mammalian infection outbreaks were reported to the World Organization of Animal Health (WOAH) from many countries, many mammalian species were affected, from pets such as cats and dogs to wild mammals, seals, sea lions, and dolphins with a dead-end infection [17, 20].

In Egypt, H5 clade 2.3.4.4b was first isolated from migratory birds in late 2016 in the form of H5N8 and was then introduced into domestic poultry and was detected in different poultry sectors such as live bird markets, backyard flocks, and commercial farms in several governorates in Egypt [21, 22]. At least six introductions of H5N8 viruses have been detected so far in Egypt with many reassortments between Eurasian, Russian and European, and Russian and Asian lineages [5, 12]. In 2021, H5N1 HPAI virus of the same clade was first detected in live bird market (LBM) in Egypt in both migratory and domestic birds [23]. With increasing dispersal and recorded cases of A(H5N1) clade 2.3.4.4b globally, the detection of A (H5N8) clade 2.3.4.4b has significantly decreased [15]. The genetic properties of the H5N8 virus haven’t been fully studied yet.

In this study, we aimed to monitor the evolution of HPAI H5N8 viruses circulating in Egypt through genetic and antigenic characterization and identify the pathogenicity and mammalian adaptation *in vitro* and *in vivo*.

## Materials and methods

### Viruses

Three H5N8 HPAI viruses, A/duck/Egypt/BA19903OP/2021 (H5N8/19903), A/common pochard/Egypt/DT19799C/2021 (H5N8/19799), and A/duck/Egypt/BA20036OP/2022 (H5N8/20036) were isolated from poultry and wild birds during 2021–2022 as part of an ongoing long-term active surveillance for avian influenza viruses as previously described [24]. A fourth H5N8 virus, A/chicken/Egypt/877OP/2016 (H5N8/877) was isolated from the first outbreak introduction in Egypt in 2016. The working virus stocks were propagated in the allantoic cavities of 10-day-old specific pathogen-free embryonated chicken eggs (SPF-ECE). After being incubated for 48 hours post-injection, eggs were chilled at 4°C for 4 hours, then the allantoic fluids were collected and pooled from ten eggs, clarified by centrifugation, and frozen in aliquots at −80°C. Virus titers were determined by 50% egg infectious dose (EID_50_) according to the method of Reed and Muench [25].

### Viral sequencing

A total of 200 μl of the clarified virus was used for viral genomic RNA extraction using the automated MagNA Pure 96 platform, KingFisher Flex instrument (Thermo Fisher Scientific, Rocklin, CA, USA), according to the manufacturer’s instructions. Whole genome sequencing was attempted on the extracted RNA from the three isolates using Illumina’s Nextera XT DNA Sample Preparation kit as previously described [23]. Complementary DNA (cDNA) was synthesized using SuperScript III first-strand synthesis kit (Invitrogen) with the Uni12 influenza primers. Multiplex PCR of all eight gene segments was conducted by using Q5 polymerase (New England Biolabs, Ipswich, MA, USA) with the Uni12/13 primers. DNA libraries were prepared using Nextera XT DNA Library Prep Kit (Illumina, San Diego, CA, USA) and sequenced via 150 bp paired-end reads by using an Illumina MiSeq personal genome system (Illumina, San Diego, CA, USA). The full viral gene segments of H5N8 viruses were assembled using CLC Genomics Workbench, version 23 (CLC Bio, Qiagen, Hilden, Germany). Sequences were submitted to GenBank.

### Genetic and phylogenetic analysis

Sequences were aligned with the reference sequences and with all H5N8 full genome sequences previously published from Egypt, obtained from the Global Initiative on Sharing All Influenza Data (GISAID) database (https://platform.epicov.org/epi3/frontend#15b26b), using BioEdit Sequence alignment editor version 7.2.5. The MEGA neighbor-joining tool was used to construct the phylogenetic trees for each segment using Kimura’s two-parameter distance model, and 1000 bootstrap replicates in the MEGA X software [26]. Amino acid sequences were further analyzed using the DNASTAR-MegAlign ClustalW multiple alignment accessory application to identify host genetic markers.

### Antigenic analysis

Antigenic characteristics of the three H5N8 viruses were assessed through Hemagglutination Inhibition (HI) assay using ferret antisera raised against clade 2.3.4.4 H5 viruses. Ferret antisera were kindly provided by Dr Richard Webby of the World Health Organization Collaborating Centre for Studies on the Ecology of Influenza in Animals and Birds. One volume of ferret sera was treated with three volumes of receptor-destroying enzyme (RDE) (Denka Seiken Co. Ltd., Tokyo, Japan), and incubated overnight at 37°C, then heat-inactivated at 56°C for 30 minutes. Nonspecific hemagglutinins were eliminated by hemadsorbing one volume of serum and one volume of 5% packed chicken red blood cells (RBCs) for 60 minutes at 4 ºC. HI tests were performed according to the World Health Organization (WHO) standard protocols with two replicates for test antisera [27].

### Viral replication kinetics analysis

Replication kinetics of the H5N8/20036, 2022 and the H5N8/877 viruses were assessed in Madin-Darby canine kidney (MDCK) and human lung epithelial cell lines (A549). Cell monolayers were grown in Dulbecco’s Modified Eagle’s Medium (DMEM) (Gibco, Life Technologies) supplemented with 5% inactivated fetal bovine serum (FBS) (Gibco, Life Technologies) and 1% antibiotic-antimycotic mixture (Gibco, Life Technologies) and grown at 37°C and 5% CO_2_. Viruses were titrated by plaque titration assay in MDCK 6-well plates [27]. A multiplicity of infection (MOI) of 0.05 of the viruses was used to infect 80–90% confluent monolayers of MDCK and A549 cells in 6-well plates. After 60 min incubation at 37°C, inoculum was removed, 3 ml of maintenance medium (DMEM supplemented with 5% bovine serum albumin (BSA) and 1% antibiotic-antimycotic) were added, then incubated at 37°C in a humidified 5% CO_2_. At 12, 24, 36, 48, and 72 hours post-infection (hpi), cell supernatants were collected as triplicates. Viral titers were determined using TCID_50_ and calculated with Reed-Muench method [25].

### Animal experiments

#### Ethics statement

All animal experiments were conducted in BSL-3 isolators under the animal experiments ethical approval number 1-4-6 obtained from the Medical Research Ethics Committee of the National Research Centre, Egypt. All challenge experiments with H5N8 HPAIV were performed under the guidelines of the Animal Care and Use Committee. Animals were anesthetized with ketamine anesthesia, and all efforts were made to minimize suffering. During the experiments, the animals would be euthanized by CO_2_ asphyxiation if they manifested severe symptoms, such as inactivity, loss of appetite, or loss of 30% or more of body weight.

#### Pathogenicity and virulence of H5 isolates in SPF chickens

A total of 21 four-week-old SPF chickens (White Leghorn egg laying breed) were obtained from Koum Osheim El Fayoum, Egypt, then divided into three groups (two infected groups and one uninfected control group). Each chick was inoculated with 10^6^ EID_50_ of the H5N8/20036 and the H5N8/877 AIVs in 200 μl, or phosphate-buffered saline (PBS), inoculum distributed to mimic the natural route of infection, intranasally, orally, and intraocularly for each chicken. After six hours post-infection (hpi), seven contact chickens were added to each infected group (donors). Oropharyngeal and cloacal swabs were collected from donors and contacts at 1, 3, 5, and 7 days post-infection (dpi). Survival and morbidity were monitored daily to 14 dpi. At 3 and 5 dpi, two donor and two contact chickens were euthanized, and organs (lungs, intestines, kidneys, and brains) were collected and divided into two parts for virological and pathological studies. Viral shedding was determined in chicken organs, 0.1 gram of each organ samples was homogenized and inoculated in SPF embryonated eggs and viral titers of each sample were determined by EID_50_ using the Reed-Muench method [25]. The second part was fixed with a 10% neutral buffered formalin solution. Tissues were embedded in paraffin, sectioned, and stained with hematoxylin and eosin (H&E) and subjected to histological examination.

#### Mammalian pathogenicity and transmission in hamsters

Viral pathogenicity and transmissibility of the H5N8/20036 (2022) virus were assessed compared to H5N8/877 virus. A total of 54 female, 12-14-week-old golden Syrian hamsters (*Mesocricetus auratus*) were divided into three groups (two infected groups and one uninfected control group), then inoculated intranasally with 10^6^ EID_50_ /30 μl of the viruses or 30 μl PBS. At 6 hpi, nine uninfected hamsters were added to each infected group as contacts. Daily body weight and temperature were recorded up to 14 dpi using a medical thermometer through cheek pouches. Nasal washes were collected from donors and contacts animals at 1, 3, 5, and 7 dpi. Three infected and three contact hamsters were euthanized on 3 and 7 dpi. Nasal turbinates, tracheas, and lungs were collected from infected and contact animals. Then, all organs were subjected to virus infectivity titration in eggs to determine the EID_50_. Infected hamster lung tissues were fixed with a 10% neutral buffered formalin solution and subjected to histopathological H&E staining and examination.

#### Pathogenicity of avian influenza H5N8 viruses in mice

A total of 42 female 10-12-week-old C57BL/6 black mice were allocated into three groups (two infected groups and one control group). Mice were inoculated intranasally with 10^6^ EID_50_ / 30 μl of H5N8/20036 (2022), H5N8/877, or PBS for the control group. Daily body weight was monitored. Three infected mice were euthanized on 3, 5, and 7 dpi. Nasal turbinates, lungs, intestines, and brains were collected and then subjected to virus infectivity EID_50_ titration in eggs. Lung tissues were subjected to histopathological H&E staining and examination.

### Histological study

Specimens of the organs were subjected to paraffin histological processing and hematoxylin and eosin (H&E) staining. The histopathological changes were graded on a scale of 0 to 4 under 100X magnification using an inverted light microscope by the same pathologist [5, 28]. An unremarkable lesion received a score of 0, minimal bronchiolar epithelial changes and minimal peribronchiolar/perivascular inflammation were scored as 1, mild multifocal bronchiolar epithelial changes and mild to moderate perivascular, peribronchiolar, and alveolar inflammation obtained a score of 2, and moderate multifocal bronchiolar epithelial changes were scored as 3. Score 4 was recorded when there were marked peri-vascular, peri-bronchial, and alveolar inflammation together with diffused bronchial epithelial alterations. The intestinal mucosal lesions and villous damage were also graded where score 0 indicated normal mucosa, score 1 denoted focal desquamation of epithelial cells, score 2 moderate epithelial injury, score 3 indicated more severe epithelial injury with eroded patches, and score 4 indicated epithelial ulceration. The histological scoring system for assessing brain injury (including cerebral microgliosis, neuronal cell apoptosis, and vascular changes) and kidney injury (including glomerular sclerosis, hemorrhage, and congestion) were also recorded on a range from 0 (no lesions) to 4 (severe lesions).

## Results

### Viruses and phylogenetic analysis

Through our ongoing surveillance, three H5N8 samples were isolated between September 2021 and March 2023 from live bird markets. The first isolate H5N8/19799 was isolated from *Aythya ferina* cloacal swabs (wild migratory bird) in November 2021. The second and third isolates H5N8/20036, and H5N8/20036 were both isolated from domestic duck oral swabs in December 2021 and January 2022 respectively. Full genomes of the three isolates were then deposited in the GenBank under the following accession numbers: H5N8/19799: OR783352 to OR783359, H5N8/19903: OR783389 to OR783396, and H5N8/20036: OR783425 to OR783432.

**Fig 1:**
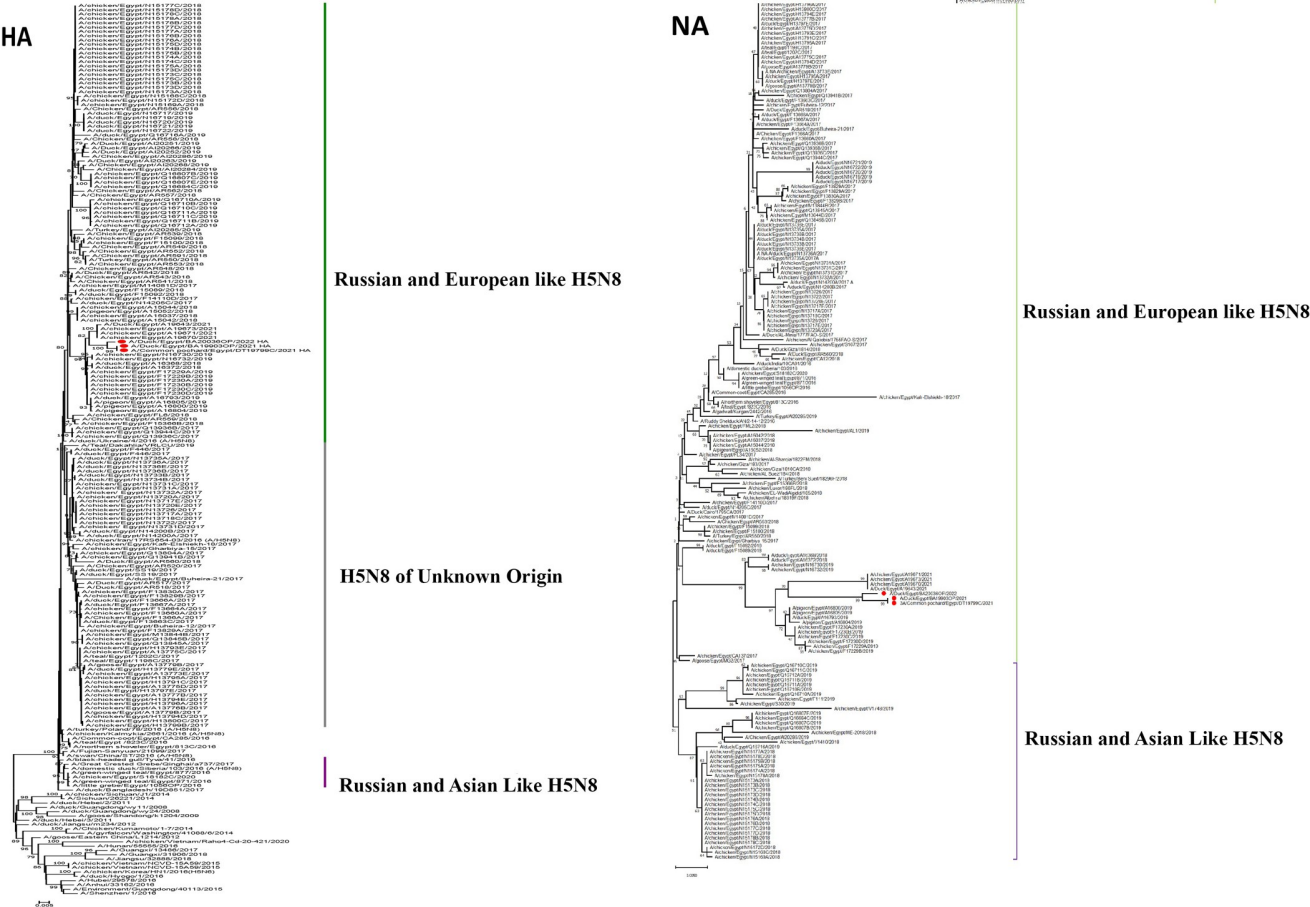
Phylogenetic trees of the HA and NA gene segments of the three H5N8 viruses (red dots) compared to previously isolated H5N8 viruses in Egypt and reference strains from different 2.3.4.4. clade. It was constructed by the neighbor-joining method with 1000 bootstraps using MEGA X software.

**Fig 2:**
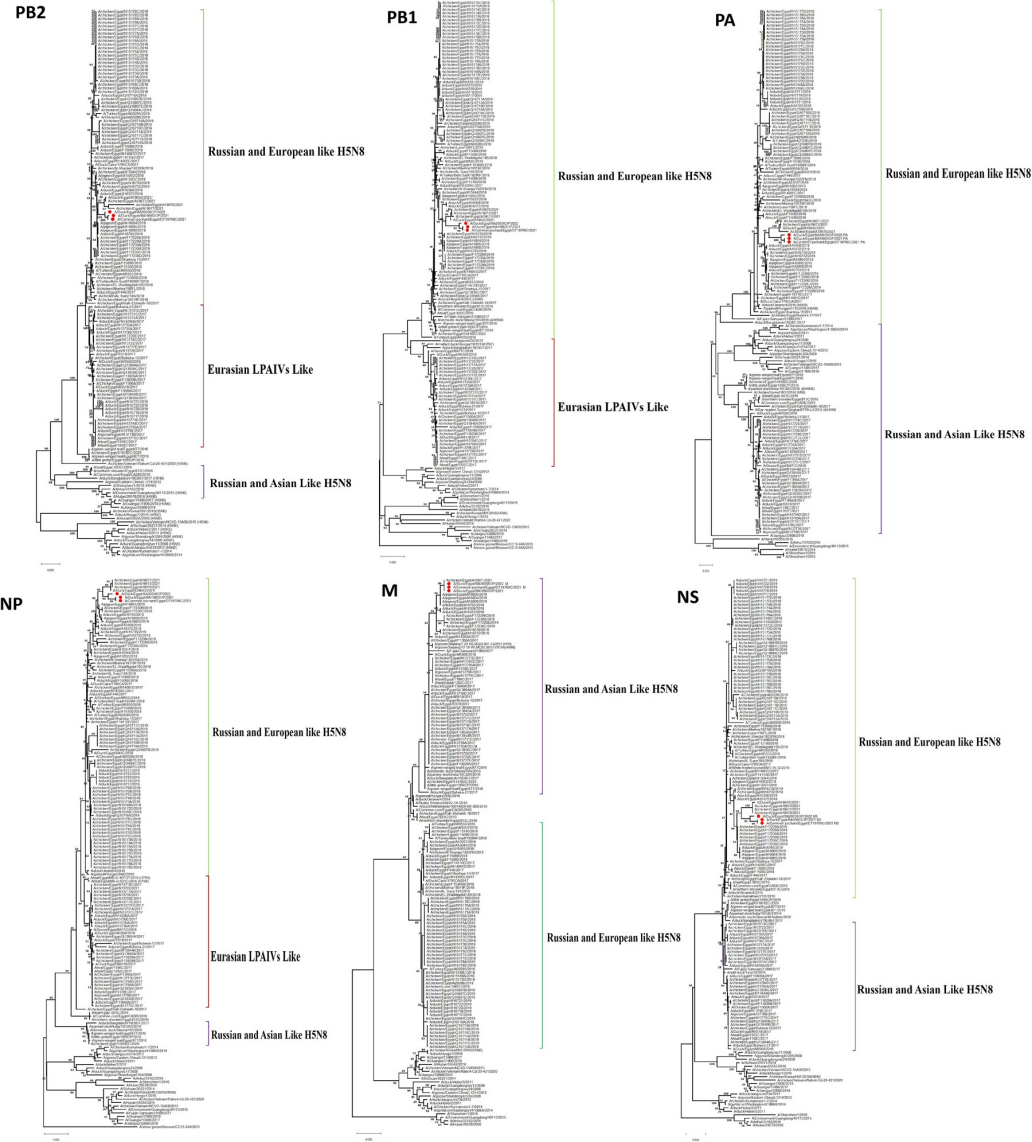
Phylogenetic trees of the six internal gene segments of the three H5N8 viruses (red dots) compared to previously isolated H5N8 viruses in Egypt and reference strains. Different genetic lineages were marked in green for Russian and European-like H5N8 viruses, Red for Eurasian-like H5N8 viruses, and purple for Russian and Asian-like H5N8 viruses.

Genetic and phylogenetic characterizations were performed on the complete gene segments after annotation using the Influenza Virus Sequence Annotation Tool, provided by NCBI Influenza Virus Resource (https://www.ncbi.nlm.nih.gov/genomes/FLU/annotation/), through which the protein Fasta file was uploaded for characterization of protein markers. The phylogenetic analysis was performed on each segment of the complete genomes obtained from the three samples in this study against previously isolated Egyptian H5N8 viruses and reference strains (Figs 1 and 2). Phylogenetic analysis of the HA sequences revealed that they belonged to the Eurasian lineage. We previously classified HA genes into three subgroups (I, II, and III) based on branch clustering of the phylogenetic tree [12]. The three isolated of H5N8 viruses in this study belong to subgroup II. Other segments, except for the M segments that showed clustering with Russian and Asian like H5N8 viruses, belonged to the Russian European lineage of H5N8. Segments of the three samples clustered together and with previous 2018 and 2019 Egyptian isolates of G4 genotype [12].

### Genetic characterization of H5N8 viruses

The analysis of the full HA gene segments of the three HPAI H5N8 viruses showed that they all belonged to phylogenetic clade 2.3.4.4b. The three HA segments showed avian-like receptor preferences (α-2,3-sialic acid linkage) in 186E, 216R, 221G, 222Q, and 224G in the hemagglutinin gene using H5 numbering [29, 30]. The multi-basic cleavage site of the HA gene was also maintained as PLREKRRKR/GLF. Analysis of the antigenic sites showed that mutations were detected in only antigenic sites A and B. All three HA sequences of H5N8 viruses had a T140A mutation in antigenic site A and a N183S substitution in the antigenic site B, while H5N8/20036 (2022) isolate had a 183N mutation. All Egyptian H5N8 isolates maintained the oseltamivir resistance mutation at 312V in NA protein [12, 31]. None of all Egyptian H5N8 isolates showed any of zanamivir or peramivir amino acid resistance markers.

PB2 segments analysis showed some mammalian replication adaptation and virulence markers such as 89V, 3098D, 318R, 356I, 477G, 495V, and 504V in the three samples similar to all previously isolated Egyptian H5N8 viruses [21, 32–34]. PB1-F2 of the three viruses’ 52 residues showed N-terminal truncation while maintaining the C-terminal essential for mitochondrial translocation and mitophagy [35]. Other mammalian adaptation and virulence residues displayed by the three isolates were PA (127V, 672L, and 550L) [12, 36, 37], NP (398Q) [38], M1 (30D and 215A) [39], and NS1 (106 M and 149A) [40, 41].

All Egyptian H5N8 displayed 43M marker in the M1 protein correlated with higher pathogenicity in infected mice, chickens, and ducks and rapid death in chickens [42]. M2 segment analysis showed that all Egyptian H5N8 viruses maintained avian preferences at residues 11T, 20S, 57Y, 78Q, and 86V [40], while displaying mammalian virulence markers at 64S and 69P [41]. None of the L26F, V27A, A30T, S31N, G34E, or L38F amantadine or rimantadine resistance markers in M2 proteins were detected in any Egyptian H5N8 isolates [43, 44].

NS1 proteins of the three isolates were 217 amino acids (aa) in length as the G3 2019 Egyptian H5N8 and lacked the PDZ motif at the C-terminal end (227GESV230) which was present in 2017 Egyptian H5N8 associated with enhanced transmission of AIVs in ferrets [45]. All Egyptian isolates also displayed 42S in NS1 protein which is responsible for mammalian adaptation, reduction of IFN-α/β expression, and controlling host antiviral immune response [46]. D189N substitution, responsible for mammalian virulence, was detected in the NS1 protein of the three isolates and was not present in isolated H5N8 viruses in Egypt before 2021 [12]. NS2 mammalian adaptation and virulence 31I marker first appeared in 2021 Egyptian H5N8 isolates and was maintained in these isolates as well [12, 47]. PA inhibitor resistance markers (E23G/K, A36V, I38T/F/M/C/L/N/S/V, E119D, and E199D/G) were absent from all Egyptian isolates.

### Antigenic analysis of H5N8 viruses

The three H5N8 isolates were tested antigenically compared to polyclonal reference serum raised against strains of H5 viruses from clades 2.3.4.4, 2.3.4.4a, H5N8 2.3.4.4b, H5N6 2.3.4.4b, 2.3.4.4c, 2.3.4.4e, 2.3.4.4f, 2.3.4.4g, 2.3.4.4h, 2.3.2.1a, 2.3.2.1c, 2.3.2.1d, and 2.2.1 as shown in Table 1. The three viruses showed the highest reactivity against (A/Astrakhan/3212/2020-like) H5N8 2.3.4.4b. No reactivity was detected against other sub-clades of 2.2, 2.3.2.1, and 2.3.4.4 confirming the genetic and phylogenetic analyses. Although both A/Common pochard/Egypt/DT19799C/2021 and A/Common pochard/Egypt/DT19799C/2021 (H5N8) showed reactivity with A/chicken/Vietnam/RAHO4-CD-20-421/2020- H5N6 2.3.4.4g, A/Duck/Egypt/BA20036OP/2022 (H5N8) antigen showed no reactivity. Despite that H5 vaccines have been used routinely in Egypt since 2006, influenza viruses evolved through antigenic drift to form new escape mutants that would show poor reactivity to commercially used vaccines from non-similar distant H5 clades [21, 48].

**Table 1:** Hemagglutination inhibition assay titers of polyclonal antibodies against different Egyptian H5N8 isolates.

Viruses / Sera	F.2015-48-A/Sichuan/26221/2014 (H5N6) 2.3.4.4a	AA/Fujian-Sanyuan/21099/2017 (CNIC-21099) H5N6 2.3.4.4b	A/gyrfalcon/WA/41088-6/2014 H5N8 2.3.4.4c	F.2019-47- A/Hubei/29578/2016 H5N6 2.3.4.4	A/duck/Hyogo/1/2016 H5N6 2.3.4.4e	A/chicken/VietNam/NCVD-15A59/2015 H5N6 2.3.4.4f.	A/Guangdong/18SF020/2018-like H5N6 2.3.4.4h	A/duck/Bangladesh/19097/2013 (SJ007) H5N1 2.3.2.1a	rg-A/duck/Vietnam/NCVD-1584/2012 H5N1 (NIBRG-301) 2.3.2.1c	2 A/chicken/Guiyang/1153/2016 (SJ009) H5N1 2.3.2.1d	A/Common magpie/Hong Kong/2256/2006 H5N1	A/Astrakhan/3212/2020-like) H5N8 2.3.4.4b	A/chicken/Vietnam/RAHO4-CD-20-421/2020- H5N6 2.3.4.4g
**F.2015-48-A/Sichuan/26221/2014 (H5N6) 2.3.4.4a**	**320**	80	160	<10	320	40	20	<10	<10	<10	<10	160	320
**AA/Fujian-Sanyuan/21099/2017 (CNIC-21099) H5N6 2.3.4.4b**	160	**40**	80	<10	160	<10	<10	<10	<10	<10	<10	80	80
**A/gyrfalcon/WA/41088-6/2014 H5N8 2.3.4.4c**	640	80	**160**	<10	320	20	<10	<10	<10	<10	<10	160	160
**F.2019-47- A/Hubei/29578/2016 H5N6 2.3.4.4**	<10	<10	<10	**160**	<10	<10	20	<10	<10	<10	<10	<10	<10
**A/duck/Hyogo/1/2016 H5N6 2.3.4.4e**	640	320	320	10	**1280**	40	<10	<10	<10	<10	<10	320	320
**A/chicken/VietNam/NCVD-15A59/2015 H5N6 2.3.4.4f.**	640	40	320	<10	1280	**80**	<10	<10	<10	<10	<10	80	40
**A/Guangdong/18SF020/2018-like H5N6 2.3.4.4h**	<10	<10	<10	<10	<10	<10	**40**	<10	<10	<10	<10	<10	<10
**A/duck/Bangladesh/19097/2013 (SJ007) H5N1 2.3.2.1a**	<10	<10	<10	<10	<10	<10	<10	**640**	1280	<10	160	<10	<10
**rg-A/duck/Vietnam/NCVD-1584/2012 H5N1 (NIBRG-301) 2.3.2.1c**	<10	<10	<10	<10	<10	<10	<10	320	**640**	<10	80	10	<10
**2 A/chicken/Guiyang/1153/2016 (SJ009) H5N1 2.3.2.1d**	<10	<10	<10	<10	<10	<10	<10	<10	<10	**20**	<10	<10	<10
**A/Common magpie/Hong Kong/2256/2006 H5N1**	<10	<10	<10	<10	<10	<10	<10	160	320	<10	**160**	10	<10
**A/Astrakhan/3212/2020-like) H5N8 2.3.4.4b**	80	40	80	<10	80	<10	<10	<10	<10	<10	<10	**80**	80
**A/chicken/Vietnam/RAHO4-CD-20-421/2020- H5N6 2.3.4.4g**	10	10	10	<10	<10	<10	<10	<10	<10	<10	<10	80	**80**
**A/duck/Egypt/BA19903OP/2021 (H5N8)**	<10	<10	<10	<10	<10	<10	<10	<10	<10	<10	<10	**80**	**80**
**A/common pochard/Egypt/DT19799C/2021 (H5N8)**	10	10	<10	<10	<10	<10	<10	<10	<10	<10	<10	**160**	**40**
**A/duck/Egypt/BA20036OP/2022 (H5N8)**	10	10	**40**	<10	<10	<10	<10	<10	<10	<10	<10	**80**	<10

### Growth kinetics in mammalian cells

**Fig 3:**
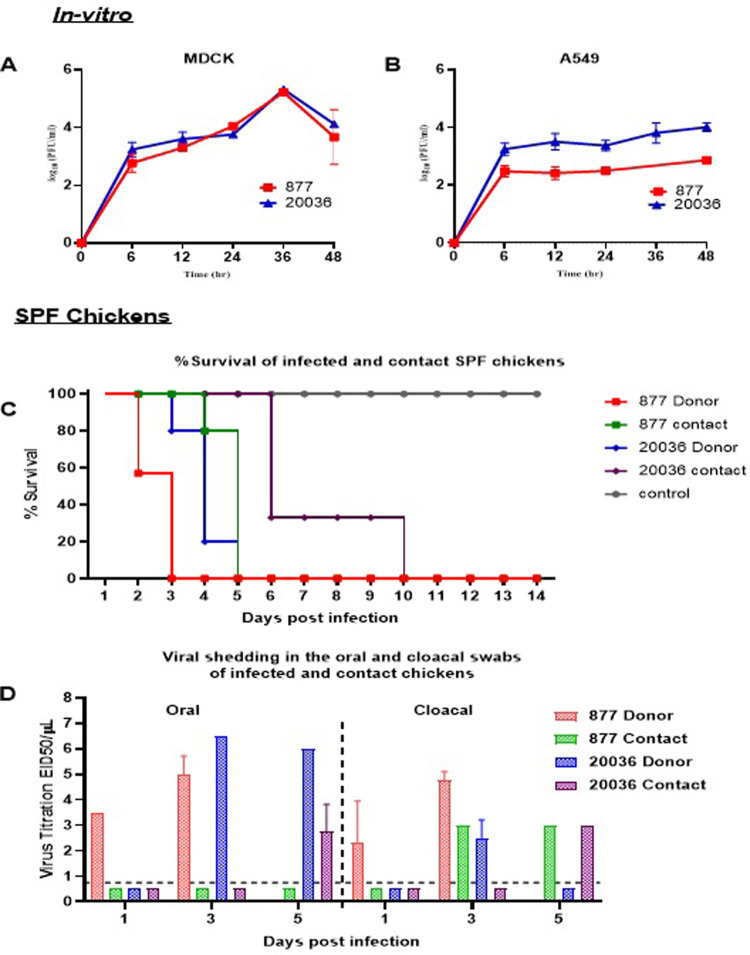
Growth kinetics of the H5N8/20036 and H5N8/877 viruses in MDCK (A) and A549 cells (B) as titrated by plaque assay. (C) Survival of H5N8 infected and contact chickens after 14 dpi. (D) Viral shedding in oral and cloacal swabs of infected and contact chickens at 1, 3, and 5 dpi.

To assess the replication kinetics, the H5N8/20036 of 2022 virus was compared to the H5N8/877 virus from the first introduction in Egypt in 2016, derived from Russian- and Asian-like H5N8 viruses. The *in-vitro* viral growth kinetics were determined in MDCK and A549 cells at an MOI of 0.05. The supernatants were collected at different time points post-infection and titrated using plaque assay, as shown in Fig 3(A) and 3(B). In MDCK, no significant difference (P>0.05) was observed between the two H5N8 viruses in the growth kinetics at different time points. Both viruses showed higher replication titers at different points in MDCK than in A549 cells. In A549, the H5N8/877 virus showed significantly lower replication titers than the H5N8/20036 virus at time points from 12 to 48 hpi.

### Replication and pathogenicity in SPF chickens

To determine the pathogenicity, replication, and transmission of H5N8 viruses, separate groups of SPF chickens were inoculated with 10^6^ EID_50_ of H5N8/20036 or H5N8/877 viruses. Then, uninfected chickens were placed in direct contact with previously infected ones as shown in Fig 3(C) and 3(D). None of the infected or contact chickens survived. The H5N8/20036 virus caused delayed mortality in both contact and infected chickens compared to the H5N8/877 virus. The onset of mortality started within 48 hpi in around 50% of chickens infected with the H5N8/877 virus and increased to 100% at 72 hpi, while H5N8/20036 infected chickens showed only 20% mortality at 3 dpi and the rest were dead at 4 and 5 dpi. the contact chickens of H5N8/877 virus showed 20% mortality at 4 dpi and the rest were dead at 5 dpi.

Viral shedding in swabs and organs was assessed by EID_50_ assay. The infected chickens with H5N8/877 virus showed positive viral shedding at 1 dpi in cloacal and oral swabs, while chickens infected with H5N8/20036 virus showed delayed viral shedding in oral and cloacal swabs at 3 dpi and oral swabs at 5 dpi. The contact chickens with H5N8/877 virus showed viral shedding in cloacal samples at 3 and 6 dpi but not in oral swabs, while contact with H5N8/20036 virus chickens showed viral shedding in oral and cloacal swabs at 5 dpi. The H5N8/877 virus also showed higher shedding titers in all tested organs in both contact and infected chickens compared to H5N8/20036 virus at 3 dpi (Table 2).

**Table 2:** Viral shedding in organs of infected and contact chickens at 3 dpi.

viruses		Viral titers (log_10_ EID_50_/mL)			
		Lung	Kidney	Brain	Intestine
**A/duck/Egypt/BA20036OP/2022**	Donor	1/2^a^ (3 ± 0) ^b^	1/2 (4 ± 0)	2/2 (6.75 ± 2.47)	2/2 (4.75 ± 0.35)
	Contact	1/2 (6 ± 0)	0/3	0/3	0/3
**A/chicken/Egypt/877OP/2016**	Donor	2/2 (6.75 ± 1.67)	2/2 (4.5 ± 0)	2/2 (3.75 ± 0.35)	2/2 (5 ± 0.7)
	Contact	2/2 (5.25 ± 2.47)	1/2 (6 ± 0)	2/2 (6.75 ± 2.47)	2/2 (3.5 ± 0.7)

^a^Number of chickens positive for virus detection /Total infected chickens.

^b^The titers are shown as the mean ± SD.

### Histopathological characterization in SPF chickens

**Fig 4:**
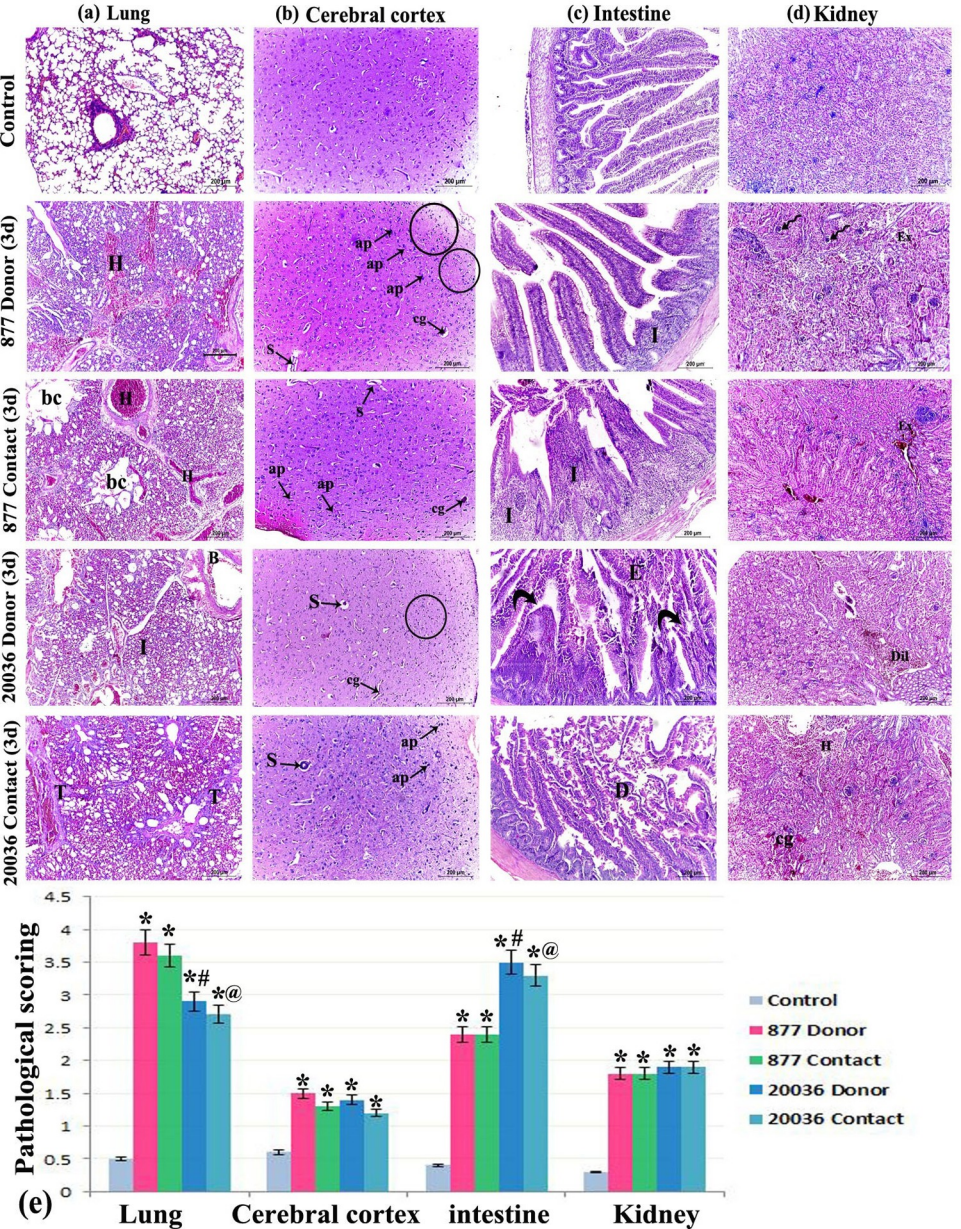
Photomicrograph of chicken tissue sections from the different experimental groups showing (a) lung sections of the control group displaying normal morphological architecture of the lung with intact lung alveoli and bronchioles. The donor chickens infected with H5N8/877 virus showed remarkable interstitial pneumonia with areas of hemorrhage (H). The contact chickens with H5N8/877 virus showed hemorrhagic pneumonia (H) with areas of collapsed alveoli and others with bronchiectasis (bc) where the alveoli appeared disrupted and irregularly-dilated. The donor chickens infected with H5N8/20036 virus showed inflammatory cellular lymphoid infiltration (I) with disruption and thickening of the bronchial wall (B). The contact chickens with H5N8/20036 showed marked thickening (T) of the inter-alveolar septa and bronchial wall. (b) The cerebral cortex of the control group showed intact neurons, neuropil, and glial cells. The donor and contact groups infected with H5N8/877 or H5N8/20036 virus showed disfigured darkly-degenerated apoptotic neurons (ap). Some capillaries appeared congested (cg) and other blood vessels showed marked dilatation of the peri-vascular space (S) (Virchow Robin’s space). The donor groups infected with H5N8/877 or H5N8/20036 virus showed marked glial cell proliferation (microgliosis) (circles). (c) The intestinal sections of the donor chickens infected with H5N8/877 virus showed inflammatory lymphoid infiltration with shortening of the villi. The contact chickens with H5N8/877 showed invasion of the mucosa and submucosa of the villi with inflammatory cells (I). The donor chickens infected with H5N8/20036 virus showed subepithelial Gruenhagen’s spaces at the tip and the sides of the villi (curved arrow) with marked shedding of the villous epithelium (E). The contact chickens with H5N8/20036 showed severe degeneration necrosis of the intestinal villi (D). (d) The kidney tissue of the control group showed normal glomeruli and tubules. The donor and contact chickens of H5N8/877 and H5N8/20036 virus showed numerous shrunken glomeruli (spiral arrows) with multiple areas of blood extravasation and inflammatory cells. The H5N8/20036 donor and contact chickens showed marked vascular dilatation and congestion (cg) with areas of hemorrhage (H) and tubular degeneration. (e) Grading of the pathological lesions observed in the different study groups. (*: significant versus control, #: significant versus H5N8/877 donor, and @: significant versus H5N8/877 contact at (P<0.05) using ANOVA, Bonferroni post hoc testing).

Organs collected from chickens at 3 dpi were subjected to fixation for routine paraffin histological processing and H&E staining (Fig 4). Examination of the lung sections of the control group displayed normal morphological architecture of the lungs with intact lung alveoli and bronchioles. The donor chickens infected with H5N8/877 virus revealed remarkable interstitial pneumonia with areas of hemorrhage. The contact chickens of H5N8/877 showed hemorrhagic pneumonia with areas of collapsed alveoli and others with bronchiectasis where the alveoli appeared disrupted and irregularly-dilated. The donor chickens infected with H5N8/20036 virus displayed inflammatory cellular lymphoid infiltration with disruption and thickening of the bronchial wall. The contact chickens with H5N8/20036 showed marked thickening of the inter-alveolar septa and bronchial wall. Evaluation of the cerebral cortex of the control group showed intact neurons, neuropil, and glial cells. The donor and contact groups of H5N8/877 or H5N8/20036 virus revealed disfigured darkly-degenerated apoptotic neurons. Some capillaries appeared congested and other blood vessels showed marked dilatation of the peri-vascular space (Virchow Robin’s space). The donor groups of H5N8/877 or H5N8/20036 virus showed marked glial cell proliferation (microgliosis).

Examination of the intestinal sections of the donor chickens of H5N8/877 showed inflammatory lymphoid infiltration with shortening of the villi. The contact chickens of H5N8/877 displayed invasion of the mucosa and submucosa of the villi with inflammatory cells. The donor chickens of H5N8/20036 showed subepithelial Gruenhagen’s spaces at the tip and the sides of the villi with marked shedding of the villous epithelium. The contact chickens of H5N8/20036 exhibited severe degeneration necrosis of the intestinal villi. Evaluation of the kidney tissue of the control group showed normal glomeruli and tubules. The donor and contact chickens of H5N8/877 revealed numerous shrunken glomeruli with multiple areas of blood extravasation and inflammatory cells. The donor and contact chickens of H5N8/20036 showed marked vascular dilatation and congestion with areas of hemorrhage and tubular degeneration (Fig 4).

### Pathogenicity and replication in BALB/C mice

**Fig 5:**
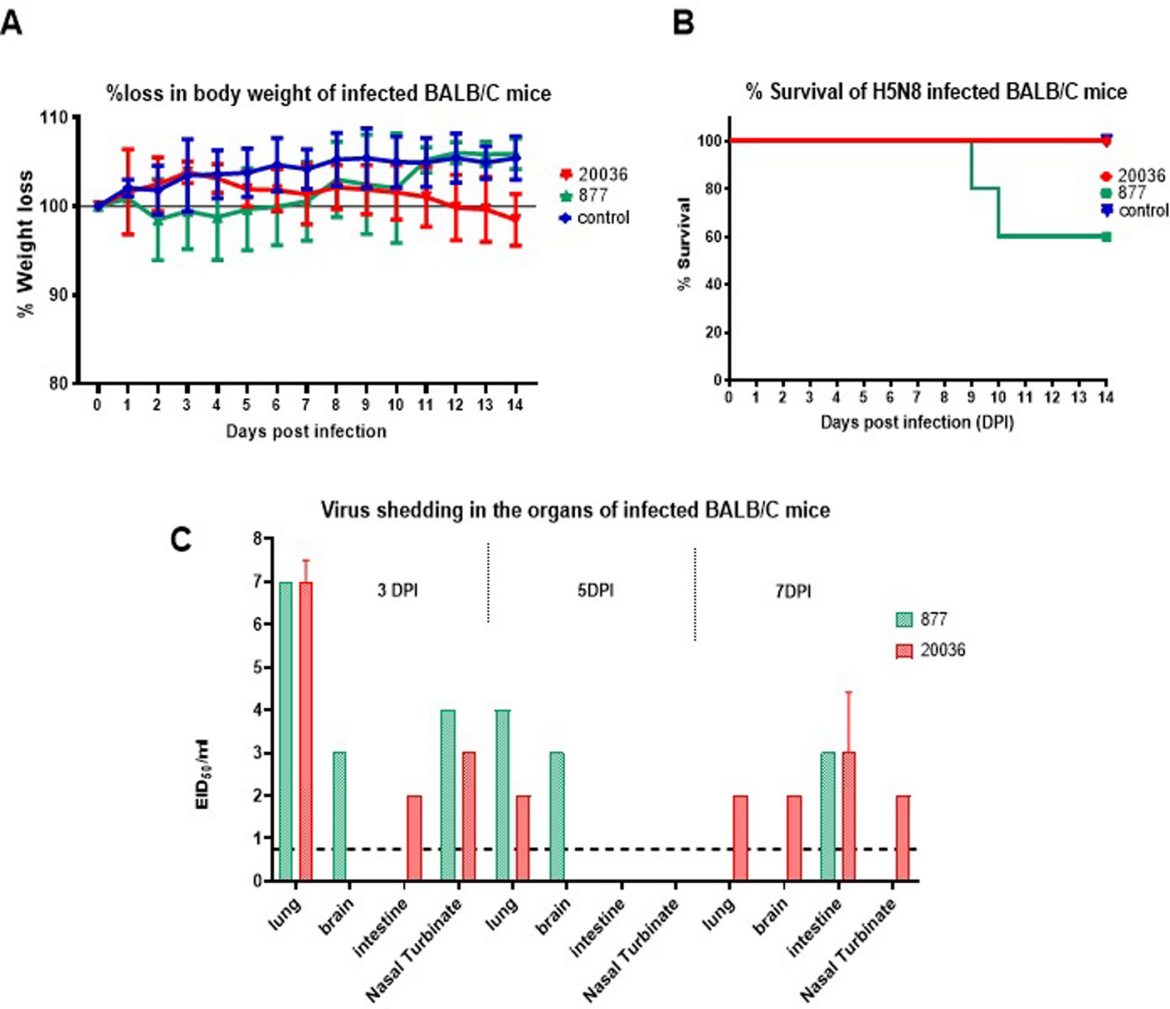
Pathogenicity and replication of H5N8 viruses in infected BALB/C mice compared to uninfected control, A) change in body weight of infected mice after 14 dpi, B) survival, and C) viral shedding of the two H5N8 viruses in infected mice organs at 3, 5, and 7 dpi.

To assess mammalian pathogenicity, replication, and adaptation, BALB/C mice groups were infected with 10^6^ EID_50_ of the H5N8/20036 or H5N8/877 viruses and then morbidity and mortality were monitored daily as shown in Fig 5. Three mice from each group were dissected at 3, 5, and 7 dpi. No significant difference in the body weight in the 3 viruses infected groups (Fig 5A) was observed. The H5N8/877 virus caused mortality in one of five mice at 9 dpi and another mouse at 10 dpi (Fig 5B). The two viruses showed systemic infection and positive viral shedding in all tested organs in at least one of the infected mice (Fig 5C). The lungs of all infected groups had positive viral shedding. The infected mice with H5N8/877 showed the highest viral shedding at 3 dpi in lungs and nasal turbinates compared to the H5N8/20036 virus. While the H5N8/877 virus showed higher pathogenicity, it had lower replication/viral shedding in mammals than H5N8/20036 virus.

### Pathogenicity and transmission in Syrian hamsters

**Fig 6:**
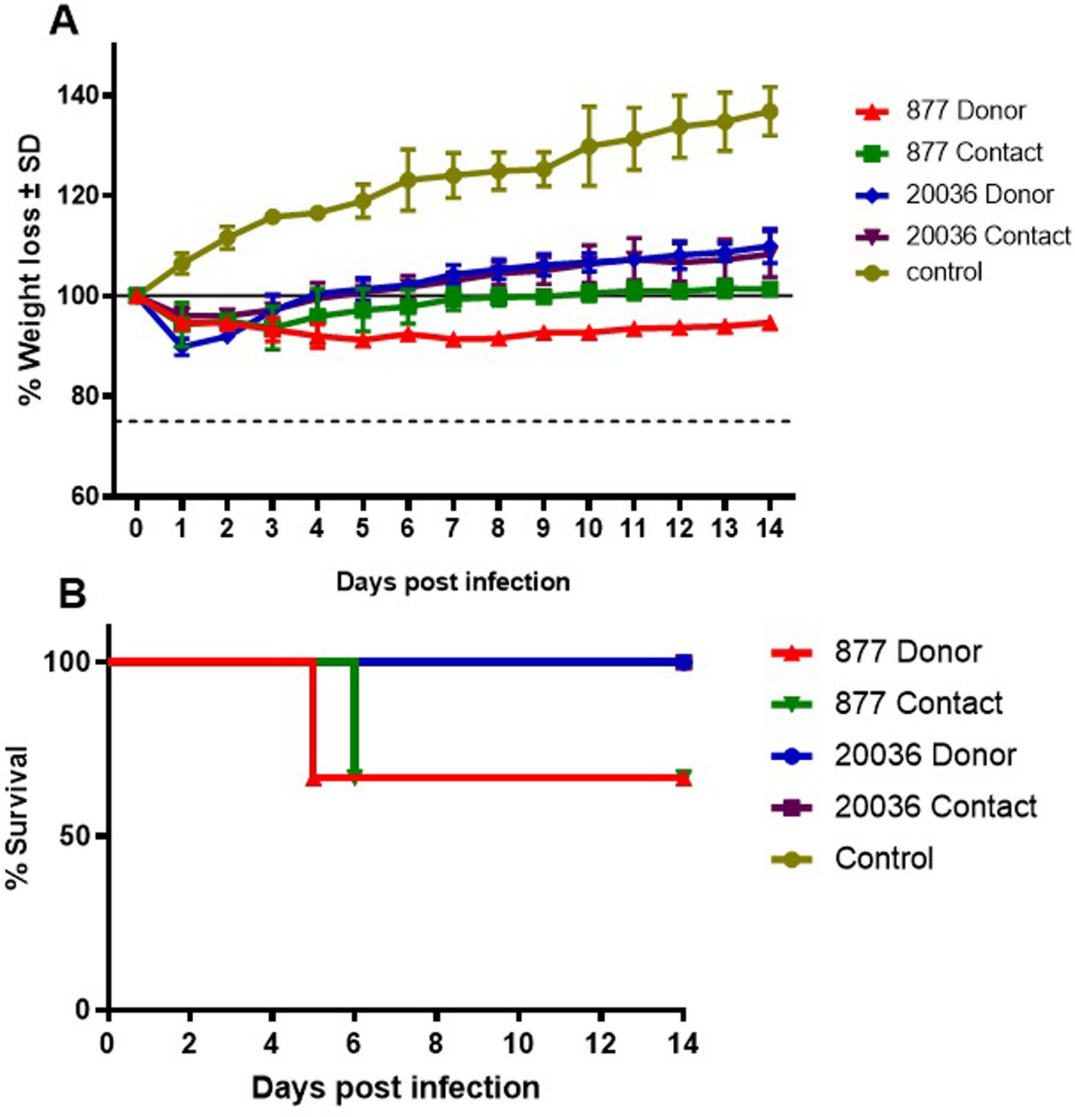
Pathogenicity and replication of H5N8 viruses in infected and contact hamsters compared to uninfected control, A) change in body weight, and B) survival rates.

The two H5N8 viruses showed varied pathogenicity in infected and contact hamsters (Fig 6). The H5N8/877 virus caused the death of one of the three infected hamsters at 5 dpi and one of the contact group at 6 dpi, while H5N8/20036 virus didn’t cause any mortality. In terms of change in body weight, no significant differences were detected between infected and contact groups, but all differed significantly from the uninfected control group. The H5N8/877 virus caused the highest loss in body weight from 4 dpi till 14 dpi. None of the infected or contact hamsters showed any significant change in body temperature.

Both viruses were detected in the nasal turbinates, tracheas, and lungs of infected animals at 3 dpi (Table 3). H5N8/877 virus showed significantly higher viral replication in lungs of infected animals at 3 and 7 dpi. All dissected infected animals with H5N8/20036 virus were positive for viral shedding in trachea and brain at 7 dpi. Positive infection of the contact hamsters and transmission through direct contact were confirmed by dissecting three contact animals at 3 and 7 dpi and testing the viral shedding in the nasal turbinates, tracheas, and lungs (Table 3). At 7 dpi, one or more hamsters showed positive viral shedding in nasal turbinates and trachea. One hamster showed positive replication in the lungs.

**Table 3:** Number, mean, and standard deviation of EID_50_ titers of the viral shedding in organs collected at 3 and 7 dpi from infected and contact Syrian hamster.

Virus	Days	Nasal Turbinate		Lung		Trachea	
		Donor	Contact	Doner	Contact	Donor	Contact
**A/chicken/Egypt/877OP/2016**	3 dpi	3/3^a^ (3.7 ± 1.26)^b^	ND	1/3 (6 ± 0)	ND	2/3 (4.25 ± 1.06)	1/3 (1 ± 0)
**A/duck/Egypt/BA20036OP/2022**		3/3 (5 ± 1.3)	1/3 (3.5 ± 0)	3/3 (1 ± 0)	ND	1/3 (2 ± 0)	ND
**A/chicken/Egypt/877OP/2016**	7 dpi	3/3 (3.5 ±2.17)	2/3 (2.25 ± 0.35)	2/3 (3.5 ±0.7)	ND	ND	2/3 (2±0)
**A/duck/Egypt/BA20036OP/2022**		2/3 (4.75 ±1.76)	1/3 (2±0)	ND	1/3 (2±0)	3/3 (3.5 ±0)	2/3 (2.75±1.06)

^a^Number of hamster positive for virus detection /Total infected hamster.

^b^The titers are shown as the mean ± SD.

ND, not detected.

### Histopathology of infected mice and hamster’s lungs

**Fig 7:**
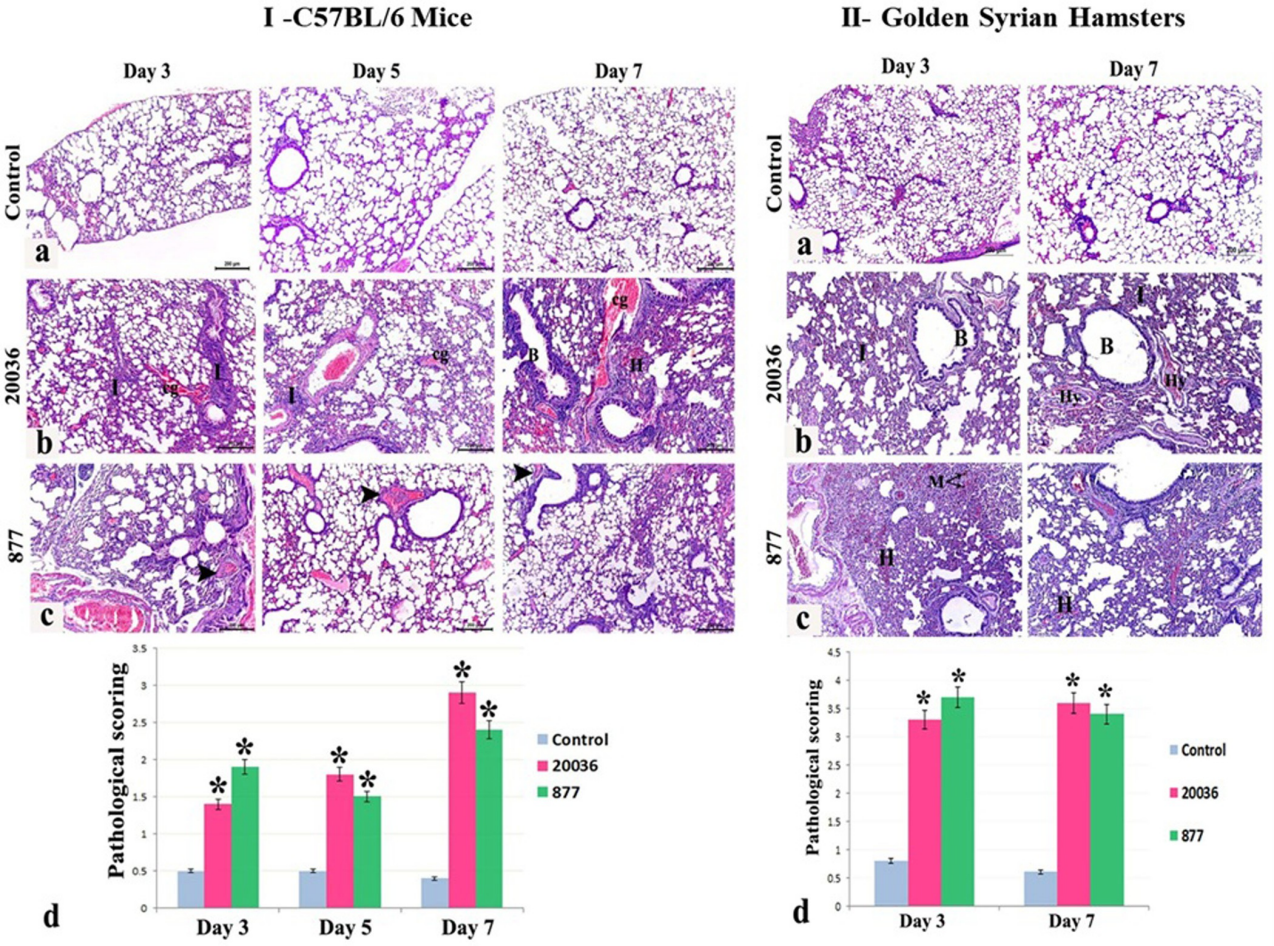
Histopathological examination of the lungs of infected mice (I) and hamsters (II). Photomicrographs of lung sections in mice from different experimental groups showed the following: (I.a) control group showing normal morphological architecture of the lung with intact lung alveoli and bronchioles. (I.b) The donor mice infected with H5N8/20036 virus showed peri-bronchial and interstitial inflammatory cellular lymphoid infiltration at days 3 and 5 (I) with vascular congestion (cg). The donor mice infected with H5N8/20036 virus showed hemorrhagic bronchopneumonia at day 7 (H), marked vascular dilatation and congestion (cg), irregularity of the bronchial walls (B), and bridging fibrosis. (I.c) The mice infected with H5N8/877 virus showed moderate interstitial pneumonia with disruption of the inter-alveolar septa and congestion of the bronchial arteries (arrowheads) at days 3, 5, and 7. (I.d) Pathological scoring of lung lesions in the different study groups. (*: Significant versus control group at (P<0.05) using ANOVA, Bonferroni post hoc testing). II) Photomicrograph of hamster lung sections from different experimental groups showed (II.a) control group with normal lung alveoli, alveolar sacs, and bronchioles. (II.b) The donor hamsters infected with H5N8/20036 virus showed inflammatory cellular lymphoid infiltration at days 3 and 7 (I) with irregularity of the bronchial wall (B) and sloughing of the bronchial epithelium. Vascular thrombosis and hyaline degeneration (Hy) were noticed at day 7. (II.c) The H5N8/877 donor hamsters infected with H5N8/877 virus showed severe hemorrhagic bronchopneumonia at days 3 and 7 (H) invading the lung parenchyma with numerous hemosiderin-laden macrophages (M) and vascular congestion. (II. d) Pathological scoring of lung lesions in the different study groups. (*: Significant versus control group at (P<0.05) using ANOVA, Bonferroni post hoc testing).

**Mice.** The lungs of infected mice at days 3, 5, and 7 (Fig 7I) and infected hamsters at days 3 and 7 (Fig 7II) were subjected to histopathological characterization. The mice lung sections of the control group showed normal morphological architecture of the lungs with intact lung alveoli and bronchioles. The donor mice infected with H5N8/20036 virus revealed peri-bronchial and interstitial inflammatory cellular lymphoid infiltration with vascular congestion at days 3 and 5. Hemorrhagic bronchopneumonia with marked vascular dilatation and congestion, irregularity of the bronchial walls, and bridging fibrosis were also observed at day 7. The mice infected with H5N8/877 virus showed moderate interstitial pneumonia with disruption of the inter-alveolar septa and congestion of the bronchial arteries at days 3, 5, and 7.

#### Hamsters

Examination of the lung sections of the control hamsters showed normal lung alveoli, alveolar sacs, and bronchioles. The donor hamsters infected with H5N8/20036 virus exhibited inflammatory cellular lymphoid infiltration with irregularity of the bronchial wall and sloughing of the bronchial epithelium at days 3 and 7. Vascular thrombosis and hyaline degeneration were noticed on day 7. The contact hamsters infected with H5N8/20036 virus revealed pneumonia, thickening of the bronchial walls, and thrombus formation at 7 days only. The donor hamsters infected with H5N8/877 virus showed severe hemorrhagic bronchopneumonia invading the lung parenchyma with numerous hemosiderin-laden macrophages at days 3 and 7. The contact hamsters with H5N8/877 virus showed disruption of the inter-alveolar septa and bronchial walls with vascular congestion at day 3 and hemorrhagic pneumonia with areas of alveolar collapse and bronchiectasis at day 7.

## Discussion

HPAIVs have been endemic to Egypt, where multiple introductions of highly pathogenic A(H5Nx) have been reported. The continued circulation of these viruses in conjunction with other avian influenza viruses within poultry populations has facilitated genetic reassortment, resulting in the emergence of novel genotypes, which can significantly impact viral evolution and pose risks to both poultry productions and public health. In the present study, we characterized the genetic properties of three H5N8 viruses isolated in 2021–2022, determined the replication kinetics in mammalians cell lines, evaluated their pathogenicity in mice model, and compared the infectivity, pathogenicity, and transmissibility in chickens and hamsters.

H5N8 viruses isolated in this study belonged to clade 2.3.4.4b and phylogenetically grouped with the 2019 viruses from G3 with high similarity to Russian and European lineages [12]. No new introductions or new genetic constellations were detected. Amino acids analysis showed that HPAI H5N8 viruses maintained the previously acquired mammalian virulence and adaptation markers. Although no human cases of H5N8 infection were reported globally including Egypt, except for the Russian outbreak of 2020 [49], such genetic changes could further affect host receptor specificity and adaptation to new hosts.

We used C57BL/6 black mice to evaluate the spill-over potential of H5N8 viruses into other mammalian models. Two viruses (H5N8/877 and H5N8/20036) used in the current study efficiently infected and replicated in mice. However, H5N8/877 virus was more pathogenic than H5N8/20036 virus which showed 20% mortality rate. Both viruses effectively replicated in all collected organs, and the highest titer was in the lung and both viruses were shed for up to 7 days. Overall, our results are consistent with previous pathogenicity studies with clade 2.3.4.4 H5N8 viruses [50–52].

We compared the infectivity, pathogenicity, and transmissibility of the H5N8 isolate (H5N8/20036) to a previous H5N8 virus from the first introduction in SPF chickens and hamsters. The H5N8/20036 virus maintained high pathogenicity but caused delayed mortality in infected chickens compared to H5N8/877 virus. Previous studies showed that clade 2.3.4.4b H5N8 viruses caused higher mortality and transmissibility in chickens [52, 53]. Both viruses transmitted to and killed all contact chickens. Oral and cloacal swabs and collected organs from both infected and contact chickens showed high titers of replication and systemic infection. Our results are consistent with previous study that compared H5N8 clade 2.3.4.4 Viruses [52]. Other studies showed that clade 2.3.4.4b has shown significant adaptation to ducks while demonstrating poor adaptation to chickens and reduced transmission [54, 55].

We used hamsters to evaluate the infectivity and transmission of H5N8 viruses in mammalian models. Several studies suggested that hamsters could serve as a useful mammalian model for influenza viruses [56–58]. Both viruses showed transmission events to close contact hamsters, as indicated by viral shedding and replication in the trachea and nasal turbinates. The H5N8/877 viral transmission could also be linked to the presence of 227G NS1 accompanied by 213S in the H5N8/877 virus that was previously linked to ferret airborne and direct contact transmission [45]. Our data indicated that Egyptian H5N8 viruses replicated successfully in mammalian cell lines and in hamsters and mice without prior adaptation and caused systematic infection. Although H5N8/877 and H5N8/20036 exhibit a closer genetic relationship, the comparative analysis of the two viruses revealed numerous amino acid variations among them. This diversity complicates the identification of molecular markers linked to enhanced infectivity and transmission in animal models. The high mortality rate in mice and hamsters infected with H5N8/877 virus compared to H5N8/20036 virus could be due to the presence of some genetic and protein markers such as the presence of NS1 PDZ binding motif. Also, the presence of PB1-F2 M46T in the H5N8/877 virus is correlated with increased mortality in mice [59].

HPAIVs The multiple introductions of 2.3.4.4b H5N8 viruses were mismatched with most of commercially used vaccines and raised the need to develop vaccines from local strains [21, 48]. As those viruses continue to evolve, antigenic compatibility of vaccine to circulating strains should be considered in countries applying or considering poultry vaccination. Our continuous surveillance showed that many genetic constellations of H5N8 were co-existing and effectively transmitting through Egyptian poultry [12].

This study emphasizes the importance of active and continuous surveillance of AIVs in both wild birds and poultry especially in settings where spill-over events could occur from wild to domestic birds and from birds to mammalian species such as live bird markets. As H5N8 viruses evolved, their pathogenicity in avian and mammalian models varied. Such monitoring is essential for evaluating viral evolution and identifying the emergence of reassortant viruses that may exhibit varying virulence in avian and mammalian hosts. It also highlights the necessity for empirical assessments of biological characteristics for continued risk assessment efforts and the effective control of HPAIV outbreaks.

## References

[1] Lin S, Chen J, Li K, Liu Y, Fu S, Xie S, et al. Evolutionary dynamics and comparative pathogenicity of clade 2.3.4.4b H5 subtype avian influenza viruses, China, 2021–2022. Virologica Sinica. 2024;39(3):358–68. Epub 2024/04/29. doi: 10.1016/j.virs.2024.04.004 ; PubMed Central PMCID: PMC11280280. 38679333 PMC11280280

[2] Xu X, Subbarao, Cox NJ, Guo Y. Genetic characterization of the pathogenic influenza A/Goose/Guangdong/1/96 (H5N1) virus: similarity of its hemagglutinin gene to those of H5N1 viruses from the 1997 outbreaks in Hong Kong. Virology. 1999;261(1):15–9. Epub 1999/09/15. doi: 10.1006/viro.1999.9820 . 10484749

[3] Organization WH. Antigenic and genetic characteristics of zoonotic influenza viruses and development of candidate vaccine viruses for pandemic preparedness. Weekly Epidemiological Record = Relevé épidémiologique hebdomadaire. 2012;87(42):401–12. 23113329

[4] Webster RG, Guan Y, Peiris M, Walker D, Krauss S, Zhou NN, et al. Characterization of H5N1 influenza viruses that continue to circulate in geese in southeastern China. Journal of Virology. 2002;76(1):118–26. doi: 10.1128/jvi.76.1.118-126.2002 11739677 PMC135698

[5] Moatasim Y, Kandeil A, Aboulhoda BE, El-Shesheny R, Alkhazindar M, AbdElSalam ET, et al. Comparative Virological and Pathogenic Characteristics of Avian Influenza H5N8 Viruses Detected in Wild Birds and Domestic Poultry in Egypt during the Winter of 2016/2017. Viruses. 2019;11(11):990. doi: 10.3390/v11110990 31717865 PMC6893538

[6] Caliendo V, Lewis NS, Pohlmann A, Baillie SR, Banyard AC, Beer M, et al. Transatlantic spread of highly pathogenic avian influenza H5N1 by wild birds from Europe to North America in 2021. Sci Rep. 2022;12(1):11729. Epub 20220711. doi: 10.1038/s41598-022-13447-z ; PubMed Central PMCID: PMC9276711. 35821511 PMC9276711

[7] van den Brand JMA, Verhagen JH, Veldhuis Kroeze EJB, van de Bildt MWG, Bodewes R, Herfst S, et al. Wild ducks excrete highly pathogenic avian influenza virus H5N8 (2014–2015) without clinical or pathological evidence of disease. Emerging Microbes & Infections. 2018;7(1):1–10. doi: 10.1038/s41426-018-0070-9 29670093 PMC5906613

[8] El-Shesheny R, Barman S, Feeroz MM, Hasan MK, Jones-Engel L, Franks J, et al. Genesis of influenza A (H5N8) viruses. Emerging infectious diseases. 2017;23(8):1368. doi: 10.3201/eid2308.170143 28609260 PMC5547793

[9] Lee Y-J, Kang H-M, Lee E-K, Song B-M, Jeong J, Kwon Y-K, et al. Novel reassortant influenza A (H5N8) viruses, South Korea, 2014. Emerging infectious diseases. 2014;20(6):1087. doi: 10.3201/eid2006.140233 24856098 PMC4036756

[10] Lee D-H, Bahl J, Torchetti MK, Killian ML, Ip HS, DeLiberto TJ, et al. Highly pathogenic avian influenza viruses and generation of novel reassortants, United States, 2014–2015. Emerging infectious diseases. 2016;22(7):1283. doi: 10.3201/eid2207.160048 27314845 PMC4918163

[11] Engelsma M, Heutink R, Harders F, Germeraad EA, Beerens N. Multiple introductions of reassorted highly pathogenic avian influenza H5Nx viruses clade 2.3. 4.4 b causing outbreaks in wild birds and poultry in The Netherlands, 2020–2021. Microbiology Spectrum. 2022;10(2):e02499–21. doi: 10.1128/spectrum.02499-21 35286149 PMC9045216

[12] Kandeil A, Moatasim Y, El Taweel A, El Sayes M, Rubrum A, Jeevan T, et al. Genetic and Antigenic Characteristics of Highly Pathogenic Avian Influenza A(H5N8) Viruses Circulating in Domestic Poultry in Egypt, 2017–2021. Microorganisms. 2022;10(3). Epub 2022/03/27. doi: 10.3390/microorganisms10030595 ; PubMed Central PMCID: PMC8948635. 35336170 PMC8948635

[13] Alkie TN, Lopes S, Hisanaga T, Xu W, Suderman M, Koziuk J, et al. A threat from both sides: Multiple introductions of genetically distinct H5 HPAI viruses into Canada via both East Asia-Australasia/Pacific and Atlantic flyways. Virus Evolution. 2022;8(2). doi: 10.1093/ve/veac077 36105667 PMC9463990

[14] Youk S, Torchetti MK, Lantz K, Lenoch JB, Killian ML, Leyson C, et al. H5N1 highly pathogenic avian influenza clade 2.3.4.4b in wild and domestic birds: Introductions into the United States and reassortments, December 2021–April 2022. Virology. 2023;587:109860. doi: 10.1016/j.virol.2023.109860 37572517

[15] Xie R, Edwards KM, Wille M, Wei X, Wong S-S, Zanin M, et al. The episodic resurgence of highly pathogenic avian influenza H5 virus. Nature. 2023;622(7984):810–7. doi: 10.1038/s41586-023-06631-2 37853121

[16] McBride D, Garushyants S, Franks J, Magee A, Overend S, Huey D, et al. Accelerated evolution of SARS-CoV-2 in free-ranging white-tailed deer. Res Sq. 2023. Epub 2023/02/25. doi: 10.21203/rs.3.rs-2574993/v1 ; PubMed Central PMCID: PMC9949239. 37640694 PMC10462754

[17] FAO W, WOAH. Ongoing avian influenza outbreaks in animals pose risk to humans 2023 [updated 12 July 2023 ]. Available from: https://www.who.int/news/item/12-07-2023-ongoing-avian-influenza-outbreaks-in-animals-pose-risk-to-humans.

[18] Osterhaus A, Mirolo M, Harder T, Beer M, Pohlmann A, Ahrens A, et al. Highly pathogenic avian influenza A virus (HPAIV) H5N1 infection in two European grey seals (Halichoerus grypus) with encephalitis. 2023. 10.1080/22221751.2023.2257810 PMC10768861 37682060

[19] Edwards KM, Siegers JY, Wei X, Aziz A, Deng Y-M, Yann S, et al. Detection of Clade 2.3. 4.4 b Avian Influenza A (H5N8) Virus in Cambodia, 2021. Emerging Infectious Diseases. 2023;29(1):170. doi: 10.3201/eid2901.220934 36573541 PMC9796211

[20] Gilbertson B, Subbarao K. Mammalian infections with highly pathogenic avian influenza viruses renew concerns of pandemic potential. Journal of Experimental Medicine. 2023;220(8). doi: 10.1084/jem.20230447 37326966 PMC10276204

[21] Kandeil A, Kayed A, Moatasim Y, Webby RJ, McKenzie PP, Kayali G, et al. Genetic characterization of highly pathogenic avian influenza A H5N8 viruses isolated from wild birds in Egypt. The Journal of general virology. 2017;98(7):1573–86. Epub 2017/07/20. doi: 10.1099/jgv.0.000847 ; PubMed Central PMCID: PMC5817258. 28721841 PMC5817258

[22] Selim AA, Erfan AM, Hagag N, Zanaty A, Samir A-H, Samy M, et al. Highly pathogenic avian influenza virus (H5N8) clade 2.3. 4.4 infection in migratory birds, Egypt. Emerging infectious diseases. 2017;23(6):1048. doi: 10.3201/eid2306.162056 28518040 PMC5443452

[23] El-Shesheny R, Moatasim Y, Mahmoud SH, Song Y, El Taweel A, Gomaa M, et al. Highly Pathogenic Avian Influenza A(H5N1) Virus Clade 2.3.4.4b in Wild Birds and Live Bird Markets, Egypt. 2022;12(1). doi: 10.3390/pathogens12010036 . 36678384 PMC9866256

[24] Kayali G, Kandeil A, El-Shesheny R, Kayed AS, Gomaa MM, Maatouq AM, et al. Active surveillance for avian influenza virus, Egypt, 2010–2012. Emerg Infect Dis. 2014;20(4):542–51. Epub 2014/03/25. doi: 10.3201/eid2004.131295 ; PubMed Central PMCID: PMC3966394. 24655395 PMC3966394

[25] Reed LJ, Muench H. A simple method of estimating fifty per cent endpoints. American journal of epidemiology. 1938;27(3):493–7.

[26] Kumar S, Stecher G, Li M, Knyaz C, Tamura K. MEGA X: Molecular Evolutionary Genetics Analysis across Computing Platforms. Molecular Biology and Evolution. 2018;35(6):1547–9. doi: 10.1093/molbev/msy096 29722887 PMC5967553

[27] Organization WH. WHO manual on animal influenza diagnosis and surveillance. World Health Organization, 2002.

[28] Zanin M, Koçer ZA, Poulson RL, Gabbard JD, Howerth EW, Jones CA, et al. Potential for Low-Pathogenic Avian H7 Influenza A Viruses To Replicate and Cause Disease in a Mammalian Model. J Virol. 2017;91(3). Epub 2016/11/18. doi: 10.1128/jvi.01934-16 ; PubMed Central PMCID: PMC5244340. 27852855 PMC5244340

[29] Matrosovich M, Zhou N, Kawaoka Y, Webster R. The Surface Glycoproteins of H5 Influenza Viruses Isolated from Humans, Chickens, and Wild Aquatic Birds Have Distinguishable Properties. Journal of Virology. 1999;73(2):1146–55. doi: 10.1128/JVI.73.2.1146-1155.1999 9882316 PMC103935

[30] Shi Y, Wu Y, Zhang W, Qi J, Gao GF. Enabling the ’host jump’: structural determinants of receptor-binding specificity in influenza A viruses. Nature Reviews Microbiology. 2014;12(12):822–31. doi: 10.1038/nrmicro3362 25383601

[31] Orozovic G, Orozovic K, Lennerstrand J, Olsen B. Detection of resistance mutations to antivirals oseltamivir and zanamivir in avian influenza A viruses isolated from wild birds. PloS one. 2011;6(1):e16028. doi: 10.1371/journal.pone.0016028 21253602 PMC3017088

[32] Li J, Ishaq M, Prudence M, Xi X, Hu T, Liu Q, et al. Single mutation at the amino acid position 627 of PB2 that leads to increased virulence of an H5N1 avian influenza virus during adaptation in mice can be compensated by multiple mutations at other sites of PB2. Virus Research. 2009;144(1):123–9. doi: 10.1016/j.virusres.2009.04.008 19393699

[33] Yamaji R, Yamada S, Le MQ, Li C, Chen H, Qurnianingsih E, et al. Identification of PB2 mutations responsible for the efficient replication of H5N1 influenza viruses in human lung epithelial cells. Journal of virology. 2015;89(7):3947–56. doi: 10.1128/JVI.03328-14 25609813 PMC4403392

[34] Shaw M, Cooper L, Xu X, Thompson W, Krauss S, Guan Y, et al. Molecular changes associated with the transmission of avian influenza a H5N1 and H9N2 viruses to humans*. Journal of Medical Virology. 2002;66(1):107–14. doi: 10.1002/jmv.2118 11748666

[35] Wang R, Zhu Y, Ren C, Yang S, Tian S, Chen H, et al. Influenza A virus protein PB1-F2 impairs innate immunity by inducing mitophagy. Autophagy. 2021;17(2):496–511. doi: 10.1080/15548627.2020.1725375 32013669 PMC8007153

[36] Llompart CM, Nieto A, Rodriguez-Frandsen A. Specific Residues of PB2 and PA Influenza Virus Polymerase Subunits Confer the Ability for RNA Polymerase II Degradation and Virus Pathogenicity in Mice. Journal of Virology. 2014;88(6):3455–63. doi: 10.1128/JVI.02263-13 24403580 PMC3957926

[37] Yamaji R, Yamada S, Le MQ, Ito M, Sakai-Tagawa Y, Kawaoka Y. Mammalian adaptive mutations of the PA protein of highly pathogenic avian H5N1 influenza virus. Journal of virology. 2015;89(8):4117–25. doi: 10.1128/JVI.03532-14 25631084 PMC4442342

[38] Chen G-W, Chang S-C, Mok C-K, Lo Y-L, Kung Y-N, Huang J-H, et al. Genomic signatures of human versus avian influenza A viruses. Emerging infectious diseases. 2006;12(9):1353. doi: 10.3201/eid1209.060276 17073083 PMC3294750

[39] Fan S, Deng G, Song J, Tian G, Suo Y, Jiang Y, et al. Two amino acid residues in the matrix protein M1 contribute to the virulence difference of H5N1 avian influenza viruses in mice. Virology. 2009;384(1):28–32. doi: 10.1016/j.virol.2008.11.044 19117585

[40] Barberis A, Boudaoud A, Gorrill A, Loupias J, Ghram A, Lachheb J, et al. Full-length genome sequences of the first H9N2 avian influenza viruses isolated in the Northeast of Algeria. Virology Journal. 2020;17(1):108. doi: 10.1186/s12985-020-01377-z 32680533 PMC7366561

[41] Kandeil A, El-Shesheny R, Maatouq AM, Moatasim Y, Shehata MM, Bagato O, et al. Genetic and antigenic evolution of H9N2 avian influenza viruses circulating in Egypt between 2011 and 2013. Archives of virology. 2014;159:2861–76. doi: 10.1007/s00705-014-2118-z 24990416 PMC4206084

[42] Nao N, Kajihara M, Manzoor R, Maruyama J, Yoshida R, Muramatsu M, et al. A Single Amino Acid in the M1 Protein Responsible for the Different Pathogenic Potentials of H5N1 Highly Pathogenic Avian Influenza Virus Strains. PLOS ONE. 2015;10(9):e0137989. doi: 10.1371/journal.pone.0137989 26368015 PMC4569272

[43] Lan Y, Zhang Y, Dong L, Wang D, Huang W, Xin L, et al. A Comprehensive Surveillance of Adamantane Resistance among Human Influenza a Virus Isolated from Mainland China between 1956 and 2009. Antiviral Therapy. 2010;15(6):853–9. doi: 10.3851/IMP1656 20834097

[44] Schnell JR, Chou JJ. Structure and mechanism of the M2 proton channel of influenza A virus. Nature. 2008;451(7178):591–5. doi: 10.1038/nature06531 18235503 PMC3108054

[45] Zanin M, Wong S-S, Barman S, Kaewborisuth C, Vogel P, Rubrum A, et al. Molecular basis of mammalian transmissibility of avian H1N1 influenza viruses and their pandemic potential. Proceedings of the National Academy of Sciences. 2017;114(42):11217–22. doi: 10.1073/pnas.1713974114 28874549 PMC5651783

[46] Jiao P, Tian G, Li Y, Deng G, Jiang Y, Liu C, et al. A Single-Amino-Acid Substitution in the NS1 Protein Changes the Pathogenicity of H5N1 Avian Influenza Viruses in Mice. Journal of Virology. 2008;82(3):1146–54. doi: 10.1128/JVI.01698-07 18032512 PMC2224464

[47] Subbarao K, Shaw MW. Molecular aspects of avian influenza (H5N1) viruses isolated from humans. Reviews in medical virology. 2000;10(5):337–48. doi: 10.1002/1099-1654(200009/10)10:5<337::aid-rmv292>3.0.co;2-v 11015744

[48] Kandeil A, Sabir JSM, Abdelaal A, Mattar EH, El-Taweel AN, Sabir MJ, et al. Efficacy of commercial vaccines against newly emerging avian influenza H5N8 virus in Egypt. Scientific Reports. 2018;8(1):9697. doi: 10.1038/s41598-018-28057-x 29946159 PMC6018731

[49] Pyankova OG, Susloparov IM, Moiseeva AA, Kolosova NP, Onkhonova GS, Danilenko AV, et al. Isolation of clade 2.3.4.4b A(H5N8), a highly pathogenic avian influenza virus, from a worker during an outbreak on a poultry farm, Russia, December 2020. Euro surveillance: bulletin Europeen sur les maladies transmissibles = European communicable disease bulletin. 2021;26(24). Epub 2021/06/19. doi: 10.2807/1560-7917.ES.2021.26.24.2100439 ; PubMed Central PMCID: PMC8212591. 34142650 PMC8212591

[50] Kim Y-I, Pascua PNQ, Kwon H-I, Lim G-J, Kim E-H, Yoon S-W, et al. Pathobiological features of a novel, highly pathogenic avian influenza A(H5N8) virus. Emerging Microbes & Infections. 2014;3(1):1–13. doi: 10.1038/emi.2014.75 26038499 PMC4217095

[51] Prokopyeva EA, Zinserling VA, Bae YC, Kwon Y, Kurskaya OG, Sobolev IA, et al. Pathology of A(H5N8) (Clade 2.3.4.4) Virus in Experimentally Infected Chickens and Mice. Interdisciplinary perspectives on infectious diseases. 2019;2019:4124865. Epub 2019/07/30. doi: 10.1155/2019/4124865 ; PubMed Central PMCID: PMC6637675. 31354812 PMC6637675

[52] Pantin-Jackwood MJ, Spackman E, Leyson C, Youk S, Lee SA, Moon LM, et al. Pathogenicity in Chickens and Turkeys of a 2021 United States H5N1 Highly Pathogenic Avian Influenza Clade 2.3.4.4b Wild Bird Virus Compared to Two Previous H5N8 Clade 2.3.4.4 Viruses. Viruses. 2023;15(11). Epub 2023/11/25. doi: 10.3390/v15112273 ; PubMed Central PMCID: PMC10674317. 38005949 PMC10674317

[53] Kwon JH, Bertran K, Lee DH, Criado MF, Killmaster L, Pantin-Jackwood MJ, et al. Diverse infectivity, transmissibility, and pathobiology of clade 2.3.4.4 H5Nx highly pathogenic avian influenza viruses in chickens. Emerg Microbes Infect. 2023;12(1):2218945. Epub 2023/06/13. doi: 10.1080/22221751.2023.2218945 ; PubMed Central PMCID: PMC10262800. 37309051 PMC10262800

[54] Puranik A, Slomka MJ, Warren CJ, Thomas SS, Mahmood S, Byrne AMP, et al. Transmission dynamics between infected waterfowl and terrestrial poultry: Differences between the transmission and tropism of H5N8 highly pathogenic avian influenza virus (clade 2.3.4.4a) among ducks, chickens and turkeys. Virology. 2020;541:113–23. Epub 2020/02/15. doi: 10.1016/j.virol.2019.10.014 . 32056709

[55] James J, Billington E, Warren CJ, De Sliva D, Di Genova C, Airey M, et al. Clade 2.3.4.4b H5N1 high pathogenicity avian influenza virus (HPAIV) from the 2021/22 epizootic is highly duck adapted and poorly adapted to chickens. The Journal of general virology. 2023;104(5). Epub 2023/05/11. doi: 10.1099/jgv.0.001852 . 37167079

[56] Iwatsuki-Horimoto K, Nakajima N, Ichiko Y, Sakai-Tagawa Y, Noda T, Hasegawa H, et al. Syrian Hamster as an Animal Model for the Study of Human Influenza Virus Infection. J Virol. 2018;92(4). Epub 2017/12/08. doi: 10.1128/jvi.01693-17 ; PubMed Central PMCID: PMC5790951. 29212926 PMC5790951

[57] Fan S, Gu C, Kong H, Guan L, Neumann G, Kawaoka Y. Influenza Viruses Suitable for Studies in Syrian Hamsters. Viruses. 2022;14(8). Epub 2022/07/28. doi: 10.3390/v14081629 ; PubMed Central PMCID: PMC9330595. 35893694 PMC9330595

[58] Shinya K, Makino A, Tanaka H, Hatta M, Watanabe T, Le MQ, et al. Systemic dissemination of H5N1 influenza A viruses in ferrets and hamsters after direct intragastric inoculation. J Virol. 2011;85(10):4673–8. Epub 2011/03/18. doi: 10.1128/JVI.00148-11 ; PubMed Central PMCID: PMC3126210. 21411541 PMC3126210

[59] Koçer ZA, Fan Y, Huether R, Obenauer J, Webby RJ, Zhang J, et al. Survival analysis of infected mice reveals pathogenic variations in the genome of avian H1N1 viruses. Scientific Reports. 2014;4(1):7455. doi: 10.1038/srep07455 25503687 PMC4264002

